# Non-syndromic Parachute Mitral Valve “When the Valve Dives in”: Case Report and Review of the Literature

**DOI:** 10.7759/cureus.52805

**Published:** 2024-01-23

**Authors:** Ahmad Damlakhy, Angelo A Messina Alvarez, Robert H Martin, Arif H Hakim, Ramegowda Rajagopal

**Affiliations:** 1 Internal Medicine, Detroit Medical Center/Sinai Grace Hospital/Wayne State University, Detroit, USA; 2 Medicine, Wayne State University, Detroit, USA; 3 Cardiology, Detroit Medical Center, Detroit, USA; 4 Cardiology, Detroit Medical Center/Sinai Grace Hospital/Wayne State University, Detroit, USA

**Keywords:** shone complex, mitral valve stenosis, mitral regurgitation, mitral valve anomaly, adult congenital heart disease, parachute mitral valve

## Abstract

A parachute mitral valve (PMV) is a congenital mitral valve anomaly diagnosed in infancy, and it can also be discovered in adults during echocardiography. Surgical management is common in infants to prevent complications from left-heart obstructions. In adults, PMV may be found independently or with other cardiac defects. Prophylactic antibiotics are recommended for certain congenital heart anomalies before dental procedures. A study suggests reconsidering guidelines to include anomalies like bicuspid aortic valve and MVP for antibiotic prophylaxis. PMV, with transvalvular blood flow turbulence, may increase the risk of infective endocarditis, as seen in a reported case with a parachute-like mitral valve. Here, we present the case of a 62-year-old female incidentally found to have a PMV during an echocardiogram.

## Introduction

Parachute mitral valve (PMV) is an extremely rare congenital cardiac defect that manifests as a unifocal attachment of the mitral valve chordae to a single, well-developed papillary muscle, typically the posteromedial papillary muscle. In contrast, the anteromedial papillary muscle is absent or hypoplastic. In the majority of patients, PMV constitutes part of the Shone complex. This complex was described more than 50 years ago and can be found in 0.6% of all congenital heart defects [1]. It is usually diagnosed in childhood and can be accompanied by mitral stenosis, subaortic stenosis, and aortic coarctation, a constellation of defects known as Shone's syndrome [2-4]. These patients require early and complex surgical management to avoid poor outcomes related to multilevel left-heart obstructions [5,6]. However, it is rare for a PMV to be newly diagnosed in adults, likely because these patients typically have either an isolated lesion or a PMV in conjunction with other milder congenital cardiac abnormalities that lead to delayed presentations [7]. We present a case of PMV that was found incidentally during echocardiography.

## Case presentation

A 62-year-old African American woman with a medical history of hypertension, Chronic Obstructive Pulmonary Disease (COPD), hypothyroidism, diabetes mellitus type II, Congestive Heart Failure (CHF), and a former tobacco user presented to the Emergency Department with persistent left-sided chest pain over the last three days. The pain, rated at 6 out of 10 in severity, was constant, stabbing, and worsened by movement. She experienced associated shortness of breath but denied other symptoms such as nausea, vomiting, sweating, difficulty breathing while lying down, lower extremity swelling, fever, chills, palpitations, syncope, or presyncope.

Normal S1 and S2 heart sounds were noted upon physical examination, with no murmurs or lower extremity edema. However, vital signs revealed an elevated blood pressure of 188/125 mmHg, a heart rate of 90 beats per minute, a respiratory rate of 16 breaths per minute, and an oxygen saturation of 97% on room air. An electrocardiogram (EKG) showed sinus rhythm without ischemic changes, and a chest x-ray appeared normal. Laboratory work-up revealed significantly elevated troponin levels of 36, 38, and 28 ng/l (normal range: 3-17 ng/l).

**Figure 1:**
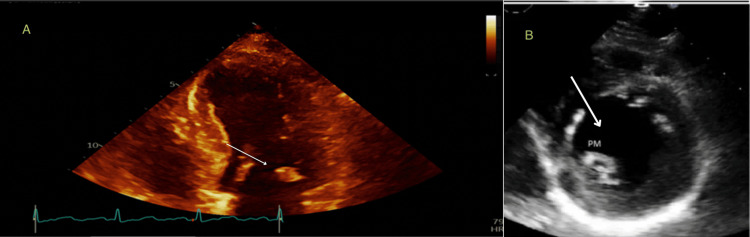
Transthoracic echocardiogram Image A: The arrow showed a small rounded mass at the tip of the posterior mitral leaflet. Image B: Parasternal short axis at the level of the papillary muscle. The arrow showed a single posteromedial papillary muscle. PM: Posteromedial

In light of concerns surrounding acute coronary syndrome, a transthoracic echocardiogram was carried out to assess the cardiac condition. The results indicated a normal ejection fraction. However, the echocardiogram also revealed a rounded mass located on the tip of the posterior mitral leaflet or chordae. This particular mass, though slightly atypical for elastoma, raised some concerns. Additionally, there was evidence of trace Mitral Regurgitation (MR) but no signs of pulmonary hypertension or any abnormality in the wall motion, as depicted in Figure 1.

**Figure 2:**
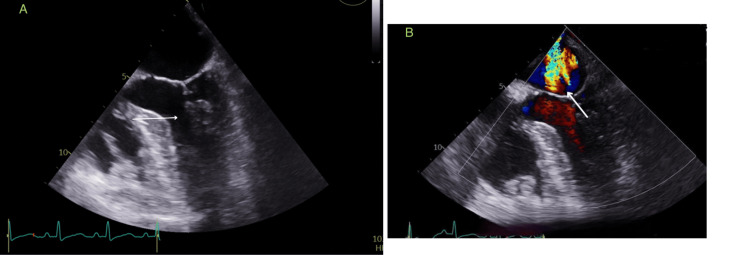
Transesophageal echocardiogram Image A: The arrow showed Posterior Mitral valve chordae originating from single papillary muscle Image B: color doppler, arrow showed significant Mitral Regurgitation

A subsequent transesophageal echocardiogram was performed to delve further into the specific pathology of the mitral valve. This more detailed investigation uncovered the presence of a congenital Posterior Mitral Valve (PMV) along with mild MR, as shown in Figure 2.

Interestingly, these findings did not fit within the criteria typically associated with Shone's complex. Importantly, the patient did not exhibit any clinical signs or symptoms indicative of CHF throughout the hospitalization period.

The patient's clinical condition exhibited a steady and marked improvement over the following days, marked by notable subsidence in chest pain with pain medication and a continual downward trend in troponin levels. As a result of comprehensive further evaluation ruling out acute coronary disease and determining the musculoskeletal nature of the chest pain (non-cardiac), it was concluded that cardiac catheterization was unnecessary. The initial elevation in troponin levels was deemed to be linked to the elevated blood pressure noted during the presentation, corroborated by the absence of wall motion abnormalities detected on the echocardiogram.

With these findings, the patient was discharged, equipped with instructions for annual echocardiographs to ensure a vigilant follow-up on her cardiac health. At present, the patient reports no adverse symptoms during her day-to-day activities.

## Discussion

PMV is a common pathological finding in the context of congenital mitral valve lesions, but it is a rare disease in adults. PMV, especially when isolated, can have an asymptomatic course into adulthood [7,8]. However, affected patients have been reported to present with symptoms of progressive dyspnea, atrial fibrillation, and even sudden death [9-12]. The lack of tell-tale symptoms typical of adult PMV means the diagnosis is often an incidental finding on transthoracic or transesophageal echocardiography performed for other reasons [9]. Indeed, echocardiography is the mainstay for diagnosing PMV, demonstrating only a single papillary muscle from which the chordae of both mitral valve leaflets are attached, aside from intra-operative findings in patients undergoing mitral valve procedures [13,14].

Regardless of how a PMV is discovered, management is driven mainly by the patient's symptoms. Severe mitral valve stenosis is the critical problem of this lesion that typically requires surgical intervention, both in children and adults, though MR can also be of concern [2,5,9]. For asymptomatic adults, no guidelines currently exist regarding how to manage these patients. In other words, it is unclear whether asymptomatic adults with incidentally discovered PMVs should undergo surgical correction at the time of diagnosis or if these patients should instead have regular cardiology follow-up with echocardiography until surgical correction is indicated. Moreover, given that PMV is an anatomical defect, no data currently exists regarding the risk of endocarditis or thromboembolic events. It is still unknown, therefore, whether these patients require prophylactic antibiotics or anticoagulation.

Moreover, as certain congenital heart anomalies necessitate the use of prophylactic antibiotics before dental procedures to prevent infective endocarditis, the use of antibiotics in other anomalies is still debatable. A study showed an increase in the incidence of infective endocarditis in patients with bicuspid aortic valve and MVP and recommended reclassifying guidelines to include these anomalies in the indications for prophylactic antibiotics [15]. PMV is associated with transvalvular turbulence of blood flow, which would account for the increased likelihood of infective endocarditis, given that there was a reported case of infective endocarditis in a parachute-like mitral valve [16].

## Conclusions

PMV is primarily a condition detected in infancy but is occasionally seen in adults. It lacks standardized management protocols. This highlights the urgent need for comprehensive longitudinal studies to explore adult PMV's long-term complications and outcomes. Concurrently, advocating for regular cardiology follow-ups, including echocardiography, is crucial to monitor and manage risks like endocarditis and thromboembolic events in adult PMV cases. Thus, emphasizing both longitudinal studies and routine cardiology check-ups is essential to bridge this medical gap and enhance care for adult PMV patients.

## References

[1] Rouskas P, Giannakoulas G, Kallifatidis A, Karvounis H (2016). Parachute-like mitral valve as a cause of mitral regurgitation. Hippokratia.

[2] Yuan SM (2020). Parachute mitral valve: morphology and surgical. Turk Gogus Kalp Damar Cerrahisi Derg.

[3] Séguéla PE, Houyel L, Acar P (2011). Congenital malformations of the mitral valve. Arch Cardiovasc Dis.

[4] Shone JD, Sellers RD, Anderson RC, Adams P Jr, Lillehei CW, Edwards JE (1963). The developmental complex of “parachute mitral valve,” supravalvular ring of left atrium, subaortic stenosis, and coarctation of aorta. The. Am J Cardiol.

[5] Nicholson GT, Kelleman MS, De la Uz CM, Pignatelli RH, Ayres NA, Petit CJ (2017). Late outcomes in children with Shone's complex: a single-centre, 20-year experience. Cardiol Young.

[6] Lee LJ, Tucker DL, Gupta S, Shaheen N, Rajeswaran J, Karamlou T (2023). Characterizing the anatomic spectrum, surgical treatment, and long-term clinical outcomes for patients with Shone's syndrome. J Thorac Cardiovasc Surg.

[7] Patsouras D, Korantzopoulos P, Kountouris E, Siogas K (2007). Isolated parachute mitral valve as an incidental finding in an asymptomatic hypertensive adult. Clin Res Cardiol.

[8] Abelson M (2001). Parachute mitral valve and a large ventricular septal defect in an asymptomatic adult. Cardiovasc J Afr.

[9] Hakim FA, Kendall CB, Alharthi M, Mancina JC, Tajik JA, Mookadam F (2010). Parachute mitral valve in adults-a systematic overview. Echocardiography.

[10] Glancy DL, Chang MY, Dorney ER, Roberts WC (1971). Parachute mitral valve: further observations and associated lesions. Am J Cardiol.

[11] Yesilbursa D, Miller A, Nanda NC (2000). Echocardiographic diagnosis of a stenotic double orifice parachute mitral valve with a single papillary muscle. Echocardiography.

[12] Fitzsimons B, Koch CG (2005). Parachute mitral valve. Anesth Analg.

[13] Liu M, Tang H (2006). Echocardiographic in the diagnosis of parachute mitral valve. Ultrasound Med Biol.

[14] Grenadier E, Sahn DJ, Valdes-Cruz LM, Allen HD, Oliveira Lima C, Goldberg SJ (1984). Two-dimensional echo Doppler study of congenital disorders of the mitral valve. Am Heart J.

[15] Friedlander AH, Couto-Souza PH (2023). Recent infective endocarditis research findings suggest dentists prescribe prophylactic antibiotics for patients having a bicuspid aortic valve or mitral valve prolapse. Med Oral Patol Oral Cir Bucal.

[16] Showkathali R, Birdi I, Khokhar A (2009). Infective endocarditis in a parachute-like asymmetrical mitral valve. Eur J Echocardiogr.

